# Single breath-hold non-contrast thoracic mra using highly-accelerated parallel imaging with a 32-element coil array

**DOI:** 10.1186/1532-429X-13-S1-P374

**Published:** 2011-02-02

**Authors:** Jian Xu, Kellyanne Mcgorty, Ruth Lim, Mary Bruno, Monvadi Srichai, Daniel Kim, Daniel K Sodickson

**Affiliations:** 1 Polytechnic Institute of New York University and Siemens Medical Solutions USA inc., New York, NY, USA; 2 Radiology of New York University, New York, NY, USA

## Purpose

To evaluate the feasibility of performing non-contrast thoracic MRA with isotropic spatial resolution within a single breath-hold.

## Background

Contrast-enhanced 3D magnetic resonance angiography (CE-MRA) provides accurate diagnosis of aortic disease [1-4]. ECG-gated CE-MRA of the thoracic aorta is challenging, due to competing demands of high spatial resolution while imaging in a narrow window of the cardiac cycle within a breath-hold. In addition, nephrogenic systemic fibrosis in patients with impaired renal function is a concern with gadolinium-based contrast agents [5]. Non-contrast ECG-gated MRA (NC-MRA) is a potential alternative [6], especially for patients with poor intravenous access or contraindications to gadolinium use. Navigator-gated NC-MRA can take approximately 10 minutes [6]. We propose to perform breath-hold, ECG-gated NC-MRA (BH NC-MRA) using highly-accelerated parallel imaging with a 32-element coil array.

## Methods

**Figure 1:**
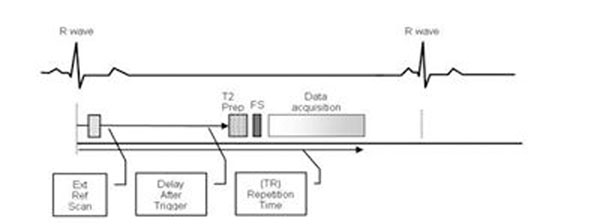
Single BH NC-MRA with the coil sensitivity and image data acquired at two different cardiac phases in the same cardiac cycle.

Following informed consent, 10 subjects (7 controls, 3 patients; 6 male, mean age=35.1 ±17.0 years) were imaged on a 1.5T scanner (Siemens, Avanto) with BH NC-MRA followed by CE-MRA. Imaging parameters for BH NC-MRA using balanced steady state free precession (b-SSFP) with T2 and fat-suppression preparation pulses were: TR/TE 2.3/1.6ms, FA70°, FOV 400x400x64mm, voxel size 1.6x1.6x1.6mm^3^, 2D GRAPPA acceleration of 3x2, segments 48, 6/8 partial Fourier in both phase encode and partition directions, partition oversampling 20%,mean scan time 19.4±4.1s. Both coil sensitivity (early systole) and MRA (mid diastole) data were acquired in the same breath hold (Figure 1). Pre- and post-contrast ECG-gated CE-MRA used similar parameters to achieve matched spatial resolution, TR/TE 3.6/1.1ms,,FA 17°,BW 330Hz/pixel,1D GRAPPA acceleration factor 2, mean scan time 39.4±10.5s. Gd-DTPA 0.15 mmol/kg at 2cc/sec was administered with arterial timing based on a timing bolus. Source and subtracted images (for CE-MRA) were reviewed in blinded fashion by a cardiologist and a radiologist. Image quality was scored (0-4; non-diagnostic to excellent) for 4 aortic segments (Table 1). Severity of artifacts was also evaluated (0-4; none to high).

**Table 1:** Comparison of image-quality and overall artifact scores between CE-MRA and NC-MRA

Aorta Segmentation	Score Mean +/- SD		Wilcoxon Test p-value
	CE-MRA	NC-MRA	
**Aortic Root^a^**	2.8 ± 0.48	2.7 ± -0.71	0.21
**Ascending Aorta^a^**	3.15 ± 0.41	2.95 ± 0.69	0.39
**Aorta Arch^a^**	3.6 ± 0.45	3.4 ± 0.61	0.2
**Descending Aorta^a^**	3.75 ± 0.26	3.7 ± 0.48	0.86
**Overall Artifact^b^**	1.1 ± 0.46	1.5 ± 0.78	0.31

## Results

**Figure 2:**
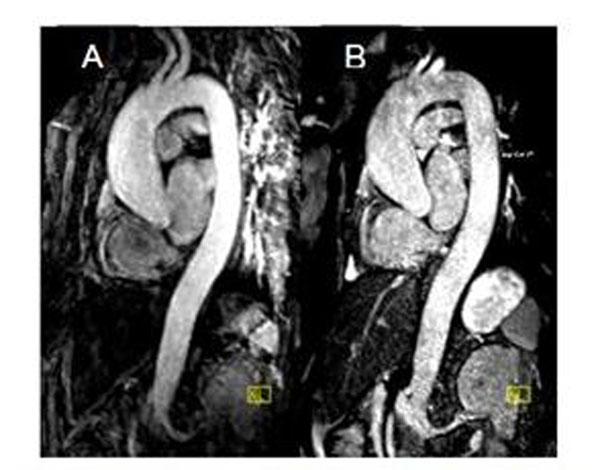
Multi planar reconstruction of A) contrast-enhanced MRA and B) noncontract enhanced MRA in a patient (59yr, Female) with aneurysm of the aortic root.

Figure 2 shows representative CE-MRA and BH NC-MRA images. For the 10 subjects studied, there was no significant difference in image quality and artifact scores (p>0.05), with diagnostic quality image scores for all evaluated segments (Table1).

## Conclusions

This study demonstrates the feasibility of performing highly accelerated single BH NC-MRA with isotropic spatial resolution and diagnostic image quality. It has potential benefits of short scan time and repeatability without need for exogenous contrast, providing rapid, safe, entirely non-invasive assessment of the thoracic aorta

## References

[B1] Gebker R Int J Cardiovasc Imaging 2007 23 6 747 756 10.1007/s10554-006-9204-6 17285264

[B2] Groves EM Am J Roentgenol 2007 188 2 522 528 10.2214/AJR.05.1467 17242264

[B3] Prince MR Am J Roentgenol 1996 166 1387 1397 10.2214/ajr.166.6.8633452 8633452

[B4] Krinsky GA Am H Roentgenol 1999 173 145 150 10.2214/ajr.173.1.10397116 10397116

[B5] Kanal E Radiology 2008 246 1 11 14 10.1148/radiol.2461071267 17855656

[B6] Srichai MB Tex Heart Inst J 2010 37 1 58 65 20200628 PMC2829812

